# Surgical Management of Hallux Extensus Following Ankle Arthrodesis: A Case Report

**DOI:** 10.7759/cureus.111968

**Published:** 2026-07-02

**Authors:** Margarita-Michaela Ampadiotaki, Christos Vlachos

**Affiliations:** 1 Department of Orthopaedics, General Hospital of Laconia, Sparta, GRC; 2 Department of Orthopaedics, IASO General Hospital, Athens, GRC

**Keywords:** case report, hallux extensus, k-wire fixation, metatarsophalangeal joint, surgical correction, z-tenotomy

## Abstract

Hallux extensus is a rare deformity of the great toe characterized by hyperextension at the first metatarsophalangeal (MTP) joint, which can cause pain, impaired gait, and difficulty wearing standard footwear. Surgical intervention is often required when conservative treatment fails.

We report the case of a 72-year-old female who developed hallux extensus six months after ankle arthrodesis with plate fixation due to osteoarthritis. The patient presented with significant pain, inability to wear shoes, and difficulty walking. Physical examination revealed flexible hyperextension of the great toe at the MTP joint, and radiographs confirmed hallux extensus without bony abnormalities.

The patient underwent a surgical procedure including Z-tenotomy of the extensor hallucis longus, dorsal release of the MTP joint, adductor tendon release, and K-wire fixation. Postoperatively, the patient was placed in a Baruk-type shoe and gradually allowed weight-bearing, followed by physical therapy.

At 8 weeks post-surgery, the patient demonstrated complete correction of the deformity, significant relief of pain, and return to normal activities, including walking in regular footwear. No recurrence was observed at the one-year follow-up.

This case highlights the effectiveness of a comprehensive surgical approach for hallux extensus, combining tendon lengthening, joint release, and stabilization. Tailoring the surgical technique to the individual patient is essential. Further studies and long-term follow-up are needed to evaluate the durability of this approach.

## Introduction

Hallux extensus is an uncommon but notable deformity of the great toe characterized by excessive dorsiflexion at the first metatarsophalangeal (MTP) joint, leading to discomfort in wearing shoes and an impaired gait. In the literature, this pathology is also referred to as floating toe, ski-jump toe, turf toe, or cock-up deformity [1].

This condition may arise from several different factors, including trauma, iatrogenic causes, neurological disorders, or as a consequence of abnormal biomechanics. Hallux extensus was initially associated with neurological conditions, especially resulting from neuromuscular imbalance following cerebrovascular events, as well as with neuromuscular disorders like Charcot-Marie-Tooth disease and ‘’drop foot’’ [2]. Moreover, other causes that lead to hallux extension deformity are injuries of the plantar plate, plaster immobilization, and surgical removal of the sesamoid complex. According to the literature, after surgical reconstruction of hallux valgus, iatrogenic extension may occur. Specifically, after scarf osteotomy, if the metatarsal is shortened more than necessary or if sesamoids are removed, the flexor hallucis longus loses its tension and extensors predominate.

Treatment options range from conservative measures to surgical correction, with surgery reserved for cases that do not respond to non-operative interventions.

In the context of ankle arthrodesis, elimination of tibiotalar motion leads to compensatory gait adaptations and redistribution of plantar pressures toward the forefoot. This may result in chronic overloading of the first ray and progressive imbalance of the tendinous forces acting on the hallux, thereby predisposing to deformity development. Although surgical correction of hallux extensus has been described with generally favorable outcomes, the literature remains limited, particularly regarding cases occurring secondary to ankle arthrodesis, which are rarely reported. To our knowledge, this is the first case reported in the literature in which a patient with hallux extensus developed the condition 6 months after ankle arthrodesis with plate and screws. This patient underwent surgical intervention to fix the deformity, which involved multiple procedures, including Z-tenotomy of the extensor, dorsal release of the MTP joint, release of the adductor tendon, and K-wire fixation.

## Case presentation

**Figure 1:**
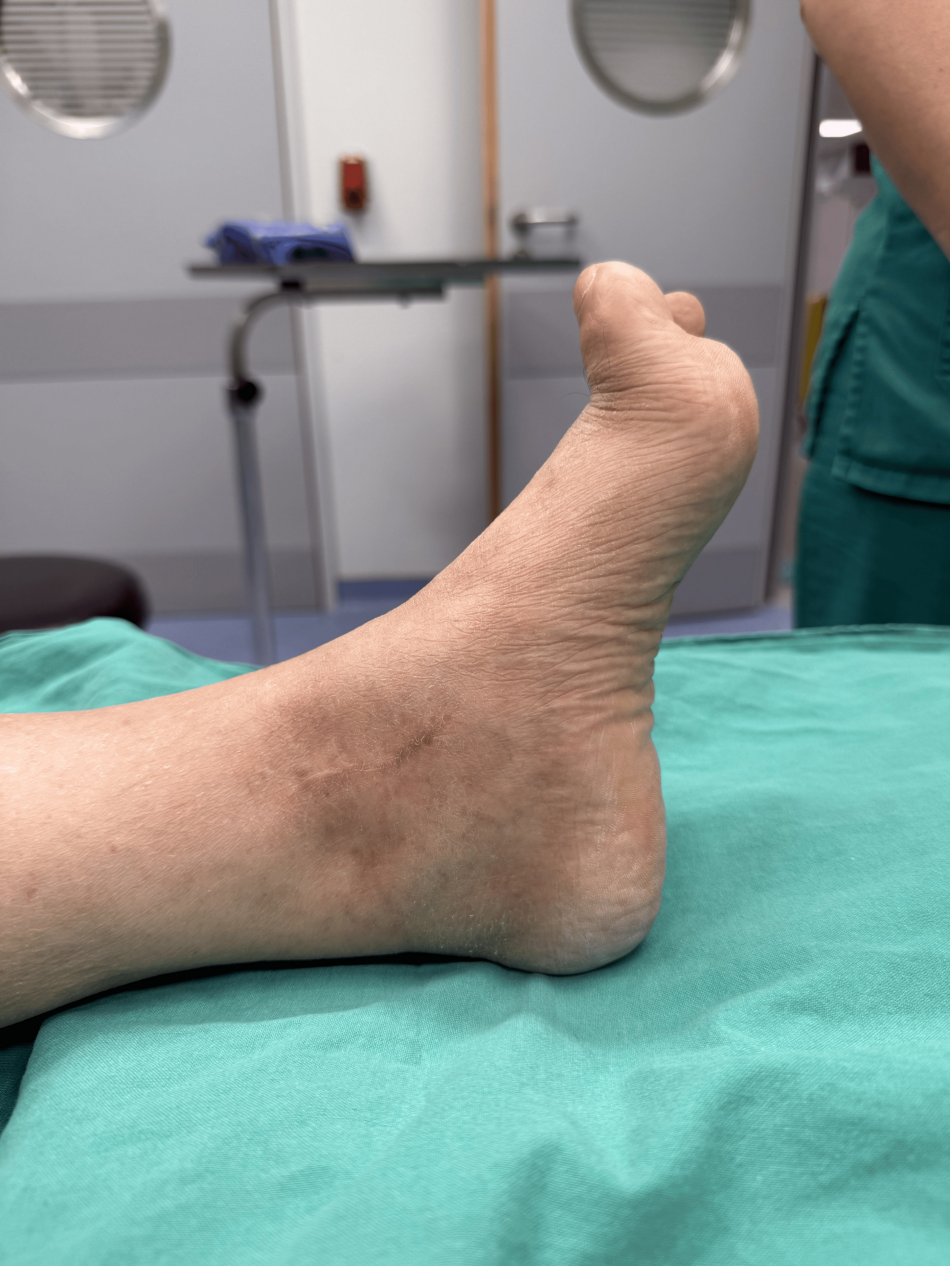
Preoperative clinical photograph demonstrating dorsal hyperextension of the hallux consistent with hallux extensus deformity.

A 72-year-old female presented to our outpatient clinic with a history of hallux extensus six months after ankle arthrodesis with a plate due to osteoarthritis, which had progressively worsened during these months (Figure 1).

The patient reported significant pain, difficulty walking, and inability to wear standard footwear due to the deformity. On physical examination, there was a noticeable dorsal deformity of the great toe with flexible hyperextension at the MTP joint. The patient had a previously performed ankle arthrodesis with a stable fusion. Neurological examination revealed no signs of peripheral neuropathy. Range of motion of the ankle joint was absent due to the arthrodesis, while subtalar and midfoot motion were within functional limits. Before surgical intervention, conservative treatment, including footwear modification, activity modification, and analgesic medication, was attempted without symptomatic relief. The patient reported progressive worsening of symptoms over time, likely related to altered gait biomechanics and increased forefoot loading following ankle fusion.

Radiographic evaluation confirmed the diagnosis of hallux extensus with no associated bony abnormalities or fractures. The decision was made to proceed with surgical correction due to the patient's failure to improve with conservative treatment.

The study was performed in accordance with the ethical principles of the Declaration of Helsinki. The patient signed a voluntary informed consent for the surgical intervention.

Surgical technique

**Figure 2:**
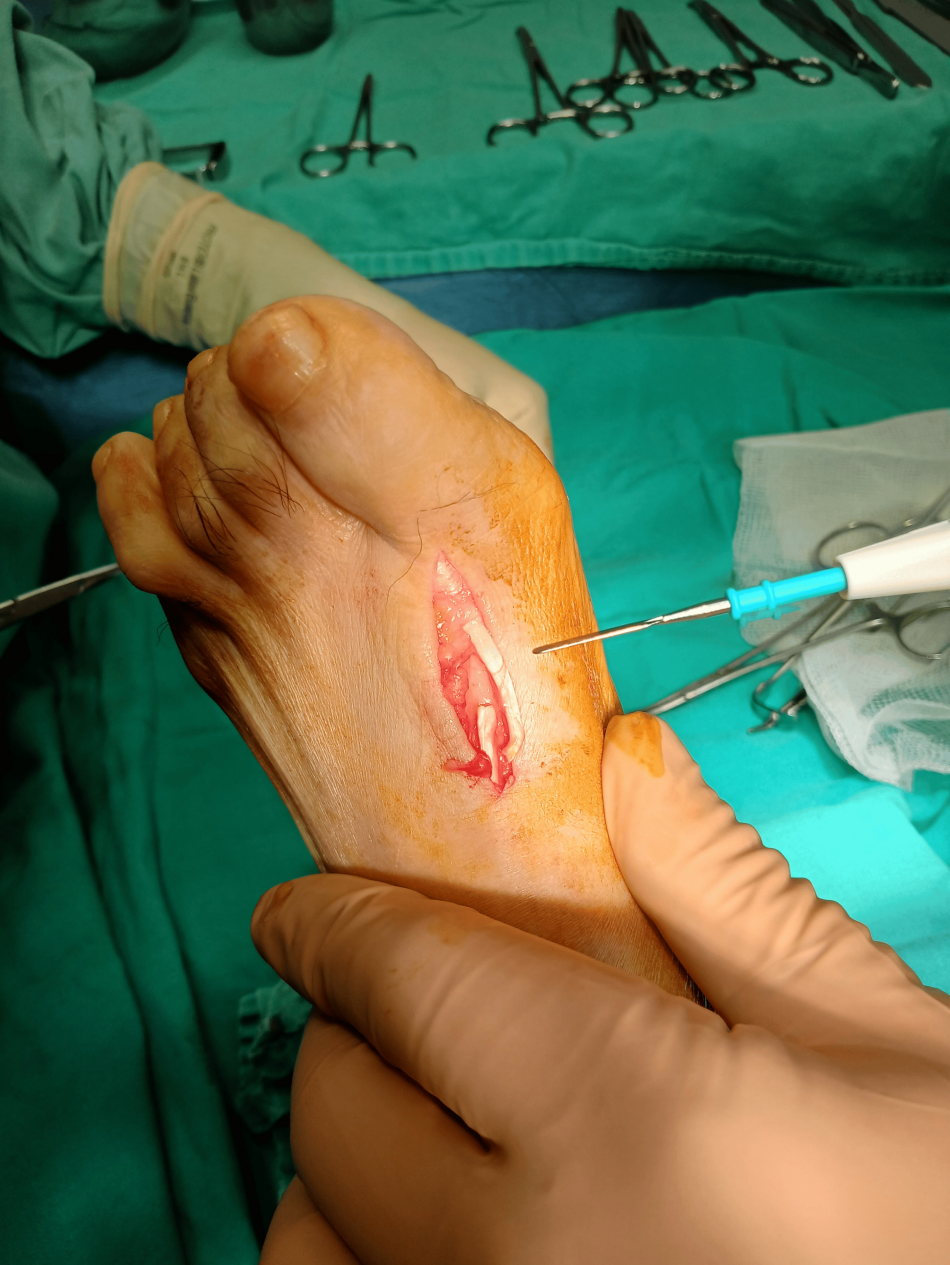
Intraoperative image showing Z-lengthening of the extensor hallucis longus tendon performed for correction of hallux hyperextension.

The operation was performed under epidural anesthesia with a tourniquet application. The patient was positioned supine, and a longitudinal incision was made approximately 4 cm proximal to the metatarsophalangeal joint, extending to the metatarsophalangeal joint. The extensor tendon was identified, and a Z-tenotomy was performed to correct the hyperextension (Figure 2).

Careful attention was given to avoid damaging the surrounding structures.

**Figure 3:**
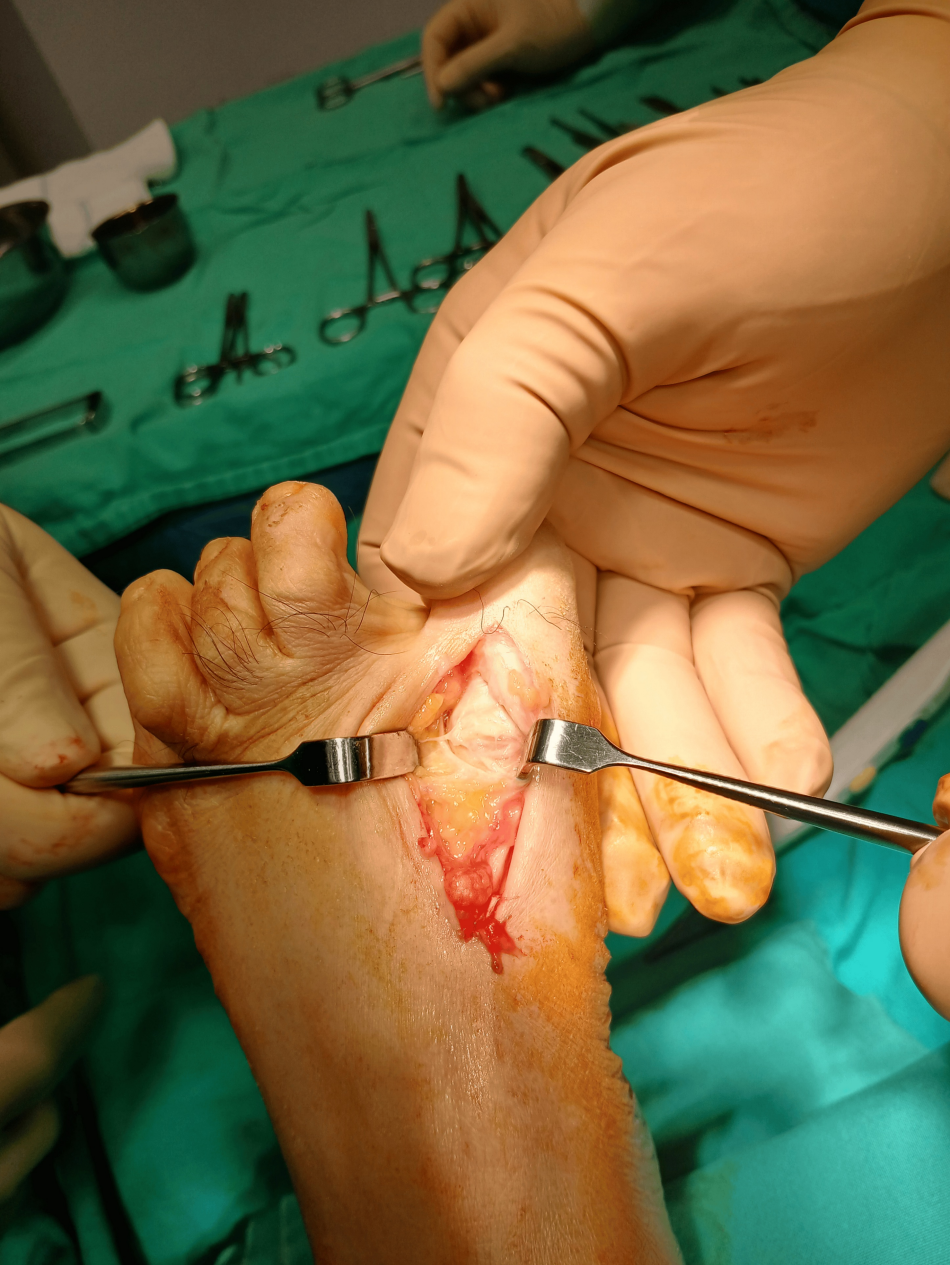
Intraoperative surgical release of the dorsal capsule of the first metatarsophalangeal (MTP) joint.

The dorsal part of the MTP joint was then released, ensuring adequate mobility for the correction of the deformity (Figure 3).

**Figure 4:**
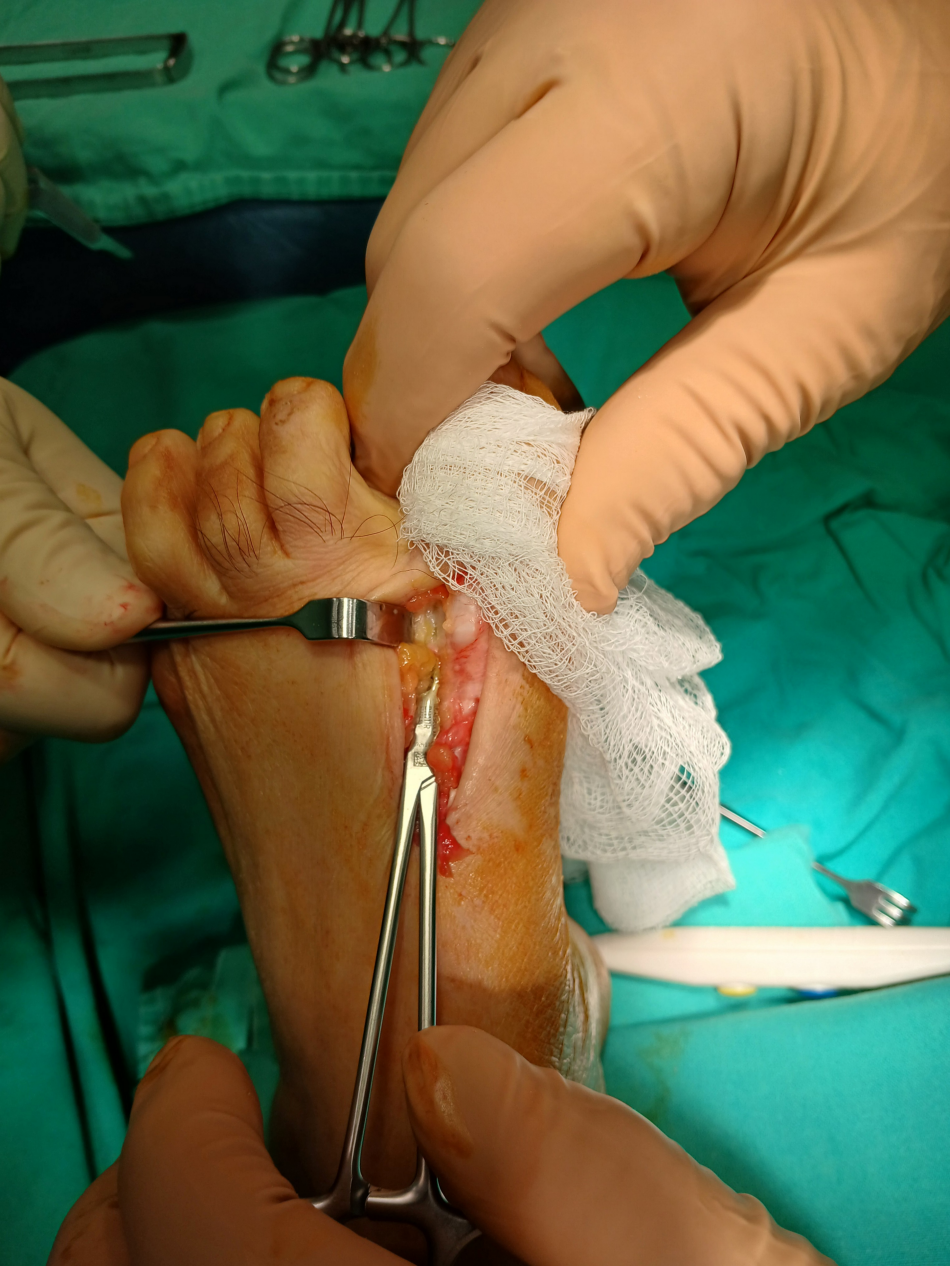
Intraoperative transection of the adductor hallucis tendon as part of the deformity correction.

The adductor tendon was transected to allow for proper alignment of the toe (Figure 4).

**Figure 5:**
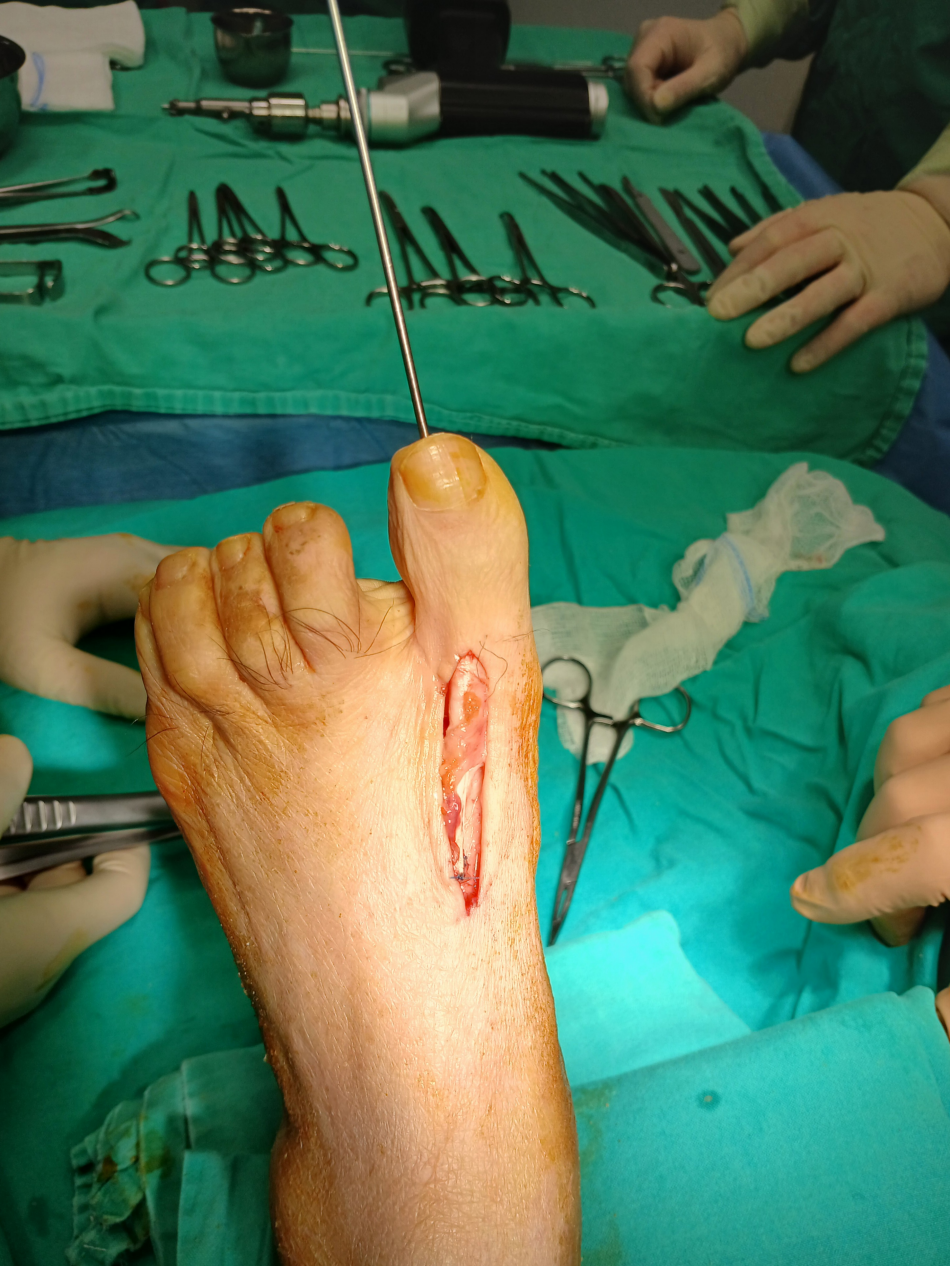
Repair of the extensor hallucis longus tendon using 3-0 nylon sutures, with temporary K-wire fixation of the first metatarsophalangeal joint.

A temporary K-wire fixation was used to stabilize the first metatarsophalangeal joint and maintain the achieved correction during the initial postoperative period until adequate soft-tissue healing occurred. Finally, the extensor tendon was sutured with 3-0 nylon sutures (Figure 5).

The wound was closed in layers, and a sterile dressing was applied.

Postoperative course

**Figure 6:**
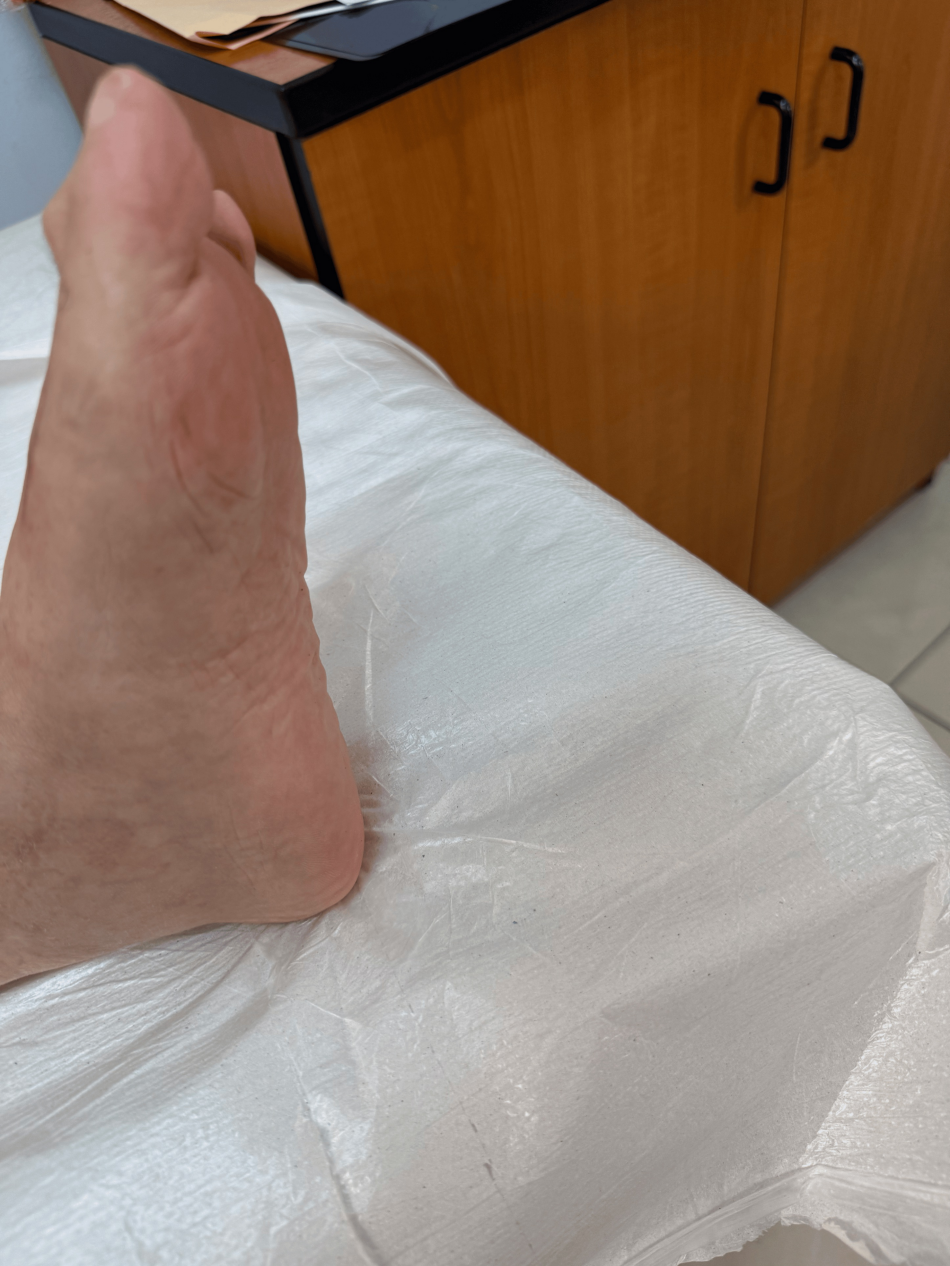
Postoperative clinical image obtained 4 weeks after surgery, demonstrating correction of the deformity.

The patient was placed in a postoperative shoe (Baruk-type shoe) and instructed to avoid weight-bearing on the affected foot for the first 4 weeks. Follow-up visits were scheduled to monitor healing and ensure proper alignment of the toe. The K-wire was removed at 4 weeks, and the patient was able to resume normal activities, including wearing shoes without discomfort (Figure 6). 

The patient was gradually allowed to increase weight-bearing, and physical therapy was recommended to restore full function. Moreover, at 6 weeks postoperatively, walking in regular shoes was allowed. At 8 weeks postoperatively, the patient showed significant improvement, with complete correction of the hallux extensus deformity and resolution of pain, while at the 1-year follow-up, she remained asymptomatic with a good clinical outcome.

## Discussion

Hallux extensus is a challenging condition to treat, and surgical intervention is often required in cases where conservative management fails. The combination of Z-tenotomy of the extensor tendon, joint release, adductor tenotomy, and K-wire fixation in this case resulted in a favorable outcome, with significant relief of symptoms and correction of the deformity.

It is essential to tailor the surgical approach to each patient's specific needs, considering factors such as the severity of the deformity, age, activity level, and overall health. Several surgical techniques have been described for the treatment and reconstruction of extensor hallucis longus pathology. These include tendon lengthening procedures such as Z-tenotomy as well as combined soft-tissue balancing approaches, which have demonstrated satisfactory outcomes in small case series. Although the technique used in this case was successful, other surgical options, such as tendon transfers or arthrodesis, may be considered in more complex cases [3,4].

In a study by de Steiger et al., four cases of hallux extensus were described. Two cases involved children aged 11 and 14 years, who were treated with osteotomy of the distal part of the proximal phalanx of the great toe and fixation with Kirschner wires. The third case concerned a 26-year-old man who was treated with arthrodesis, while the fourth case involved an 11-year-old boy with juvenile chronic arthritis who was also treated by arthrodesis. No recurrence was observed during a 5-year follow-up period [1]. Wu et al. reported a case of hallux extensus after fracture of the distal tibia and fibula, which was treated with a plate 20 years ago. In that case, Z-lengthening of the abductor hallucis tendon and the extensor hallucis longus (EHL) was performed [5]. Petrosyan A.S. also recommends correcting iatrogenic hallux extensus by either a minimally invasive percutaneous tenotomy of the long extensor tendon or, alternatively, an open Z-shaped lengthening of the long extensor tendon combined with tenotomy of the short extensor tendon [6]. McGowan D.D. likewise recommends tenotomy of the long extensor tendon of the great toe at the level of the interphalangeal joint as the preferred method for correcting hallux extensus [7]. 

In another study, the surgical outcomes of 22 patients with hallux extensus treated were analyzed. Patients were divided into two equal groups according to the surgical technique used. One group underwent percutaneous needle tenotomy of the extensor hallucis tendon, while the second group was treated with tenodesis of the musculotendinous portion of the short flexor of the great toe using the authors’ technique. Outcomes were evaluated based on patient satisfaction, radiographic findings, and American College of Foot and Ankle Surgeons (ACFAS) scores. Recurrence or inadequate correction occurred in 54% of patients treated with percutaneous tenotomy, whereas the tenodesis group demonstrated 100% good functional, pain-free, and aesthetic results with no recurrences. Overall, tenodesis of the short flexor showed superior effectiveness compared to extensor tendon tenotomy [8]. Junk and Kupka reported the case of a 17-year-old soccer player who developed a progressive dynamic hallux extensus after intramedullary nailing for a tibial and fibular fracture [9]. Although compartment syndrome did not occur, postoperative scarring and shortening of the extensor hallucis longus due to interaction with the osteosynthesis material were identified as the cause. Z-lengthening of the extensor hallucis longus tendon was performed with a good clinical outcome, with an anatomical variation of the muscle noted intraoperatively and considered essential to surgical success. 

One potential cause of hallux extensus that may be considered is anterior compartment syndrome; however, this was not present in our case. According to the existing literature, hallux extensus may be caused by adhesions, neurological disorders, ischemia, or necrosis [9]. Another proposed mechanism is entrapment of the tendon resulting from the force of a fracture or from surgical intervention [5]. In our case, careful examination of the tendon pathway revealed that it was free along its entire course. In the present case, hallux extensus may be related to altered foot biomechanics following ankle arthrodesis. By eliminating normal hindfoot motion and altering plantar pressure distribution, ankle arthrodesis can increase forefoot loading and induce compensatory biomechanical changes, which may eventually disrupt the balance of forces acting on the hallux and contribute to deformity development.

## Conclusions

This case suggests that a multi-faceted surgical approach may be beneficial in the management of hallux extensus. The combination of Z-tenotomy, joint release, adductor tendon release, and temporary K-wire fixation was associated with satisfactory functional and cosmetic outcomes in this patient. However, these findings are based on a single case with a short follow-up, and further studies with larger patient series and a longer follow-up are required to better evaluate the durability of this surgical approach.
